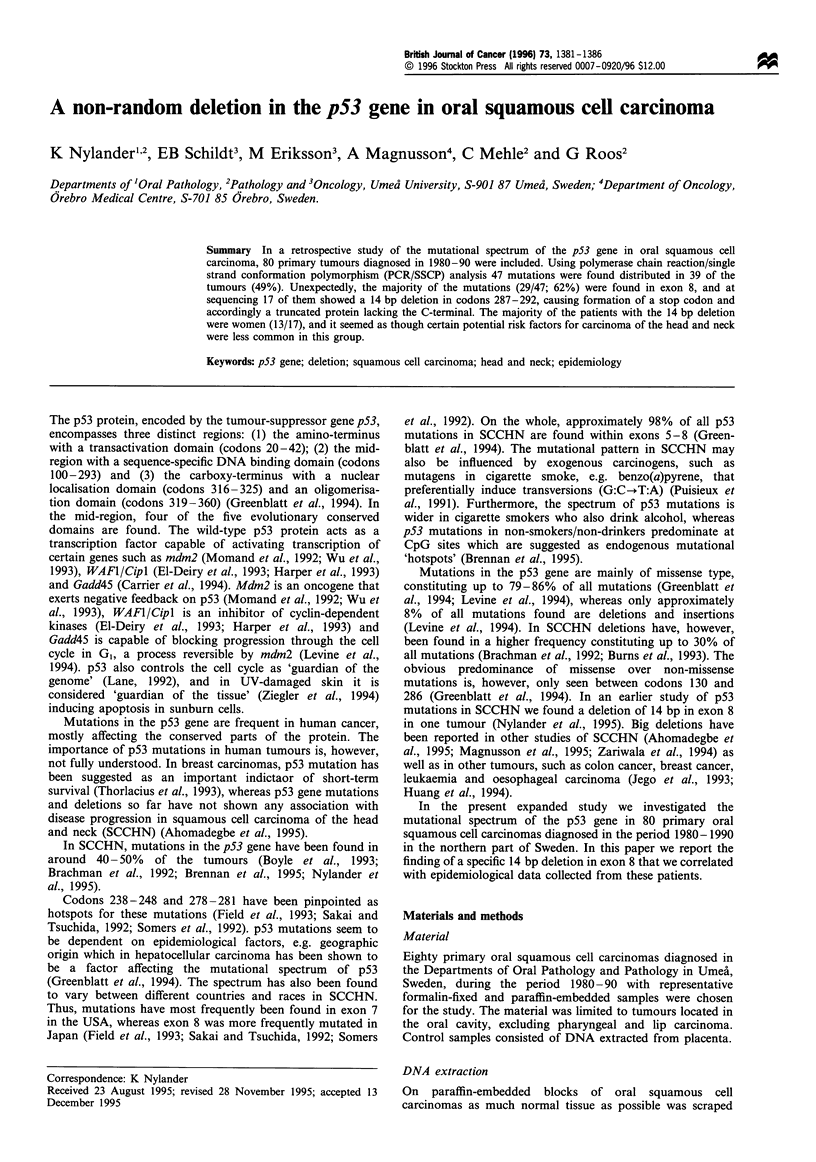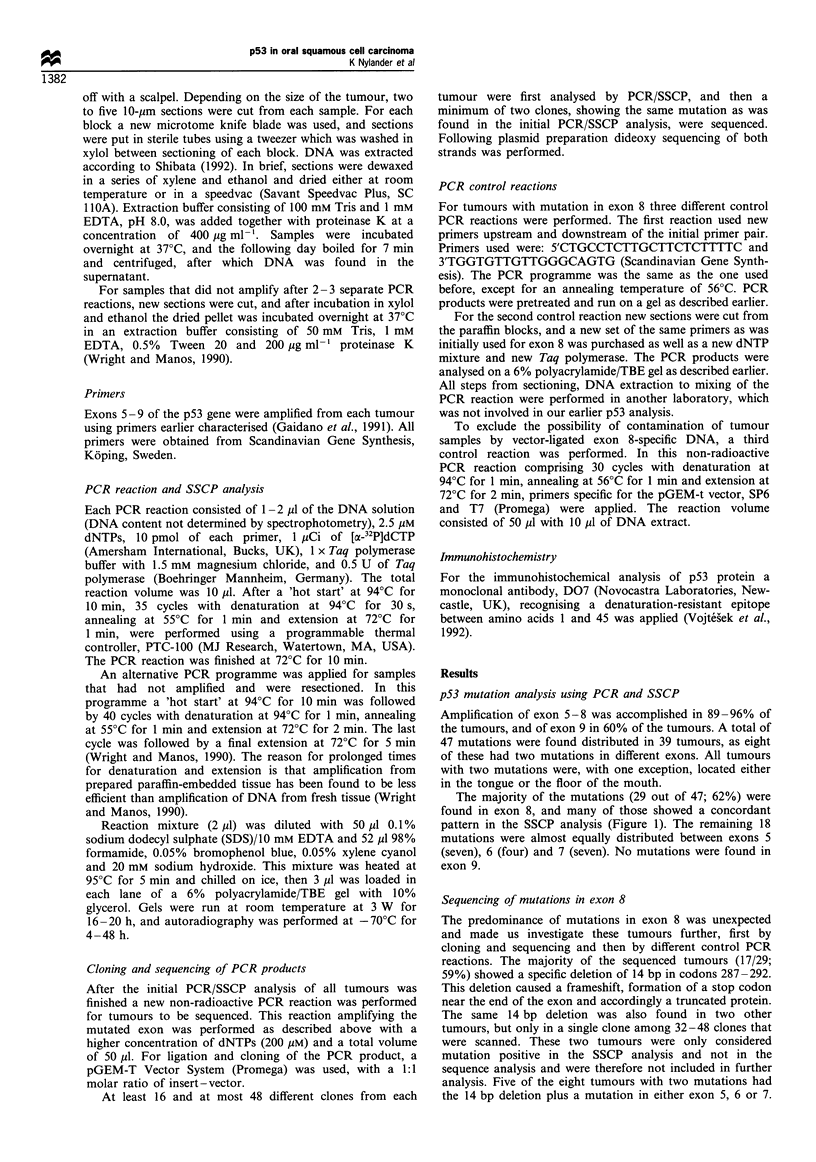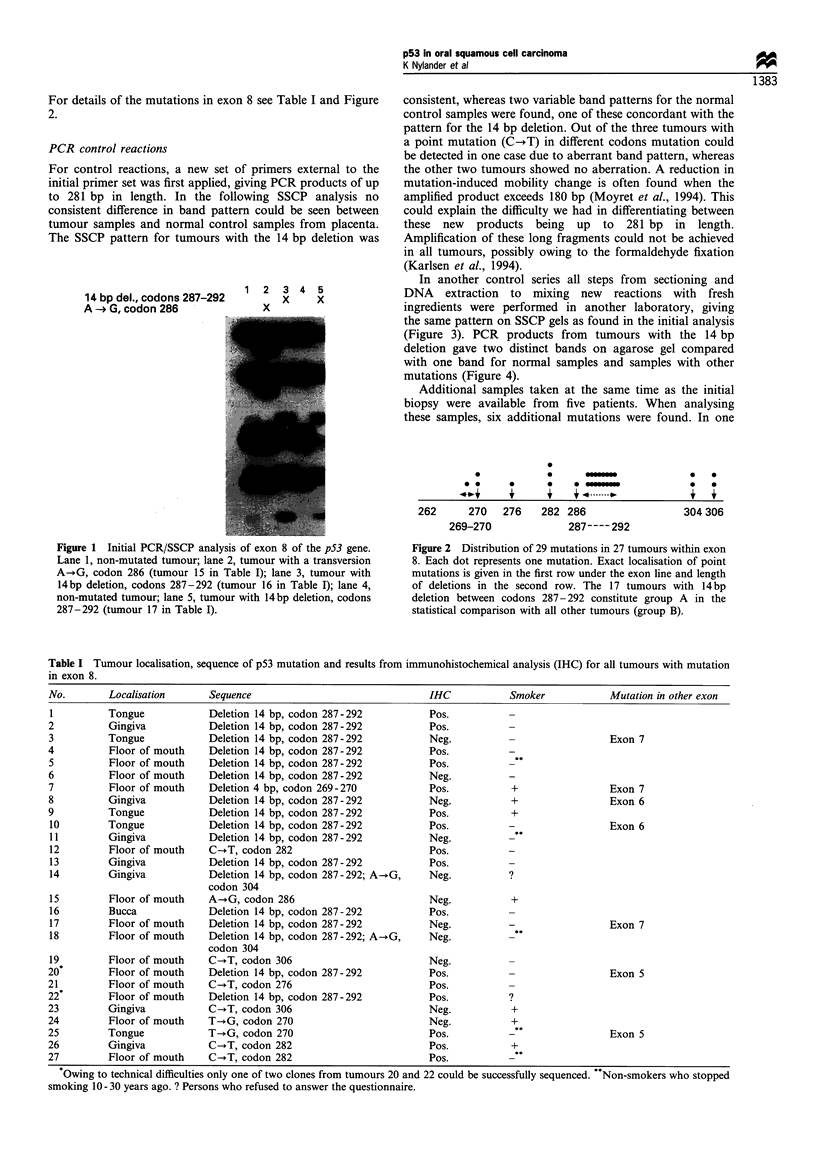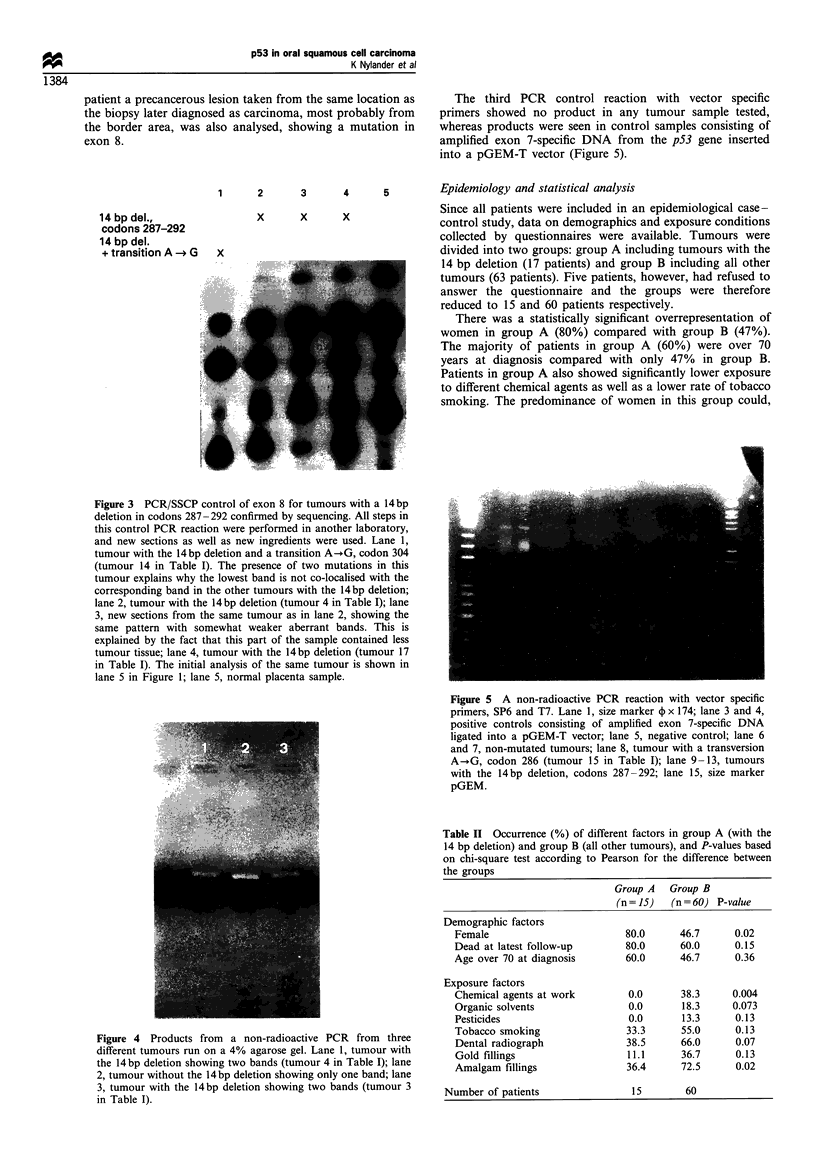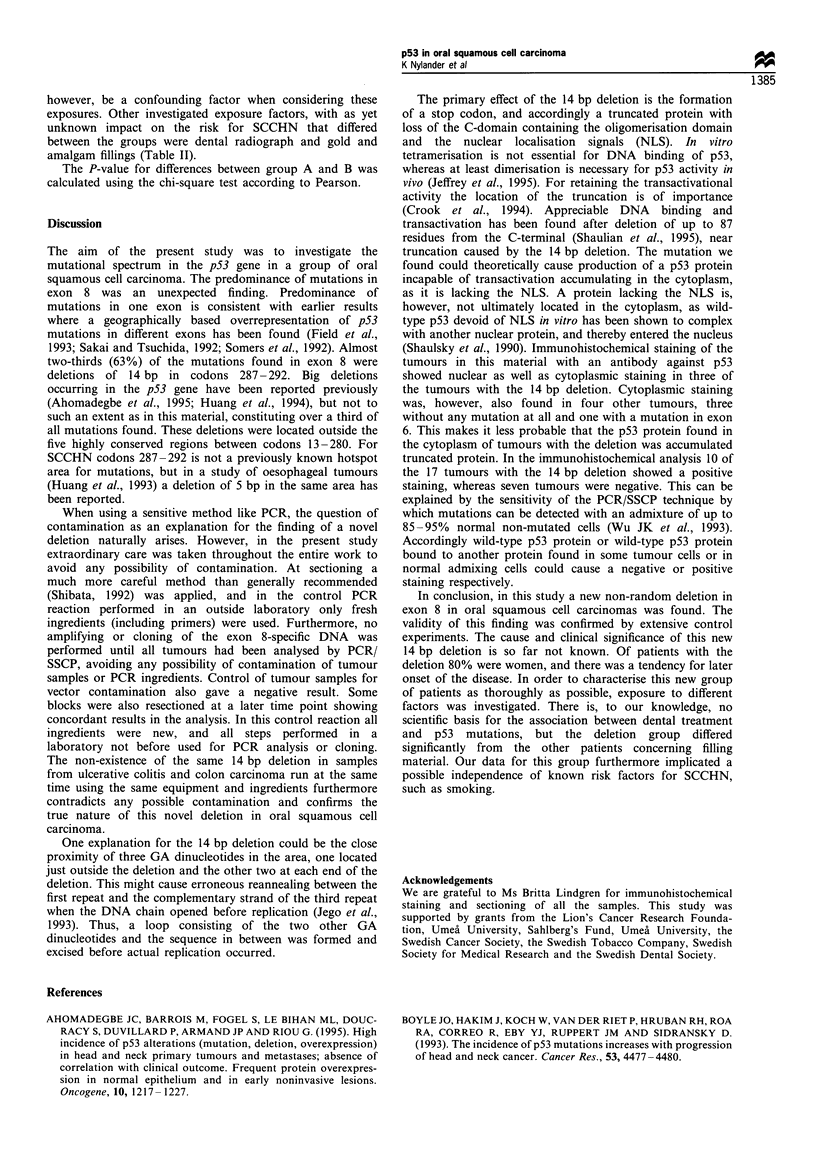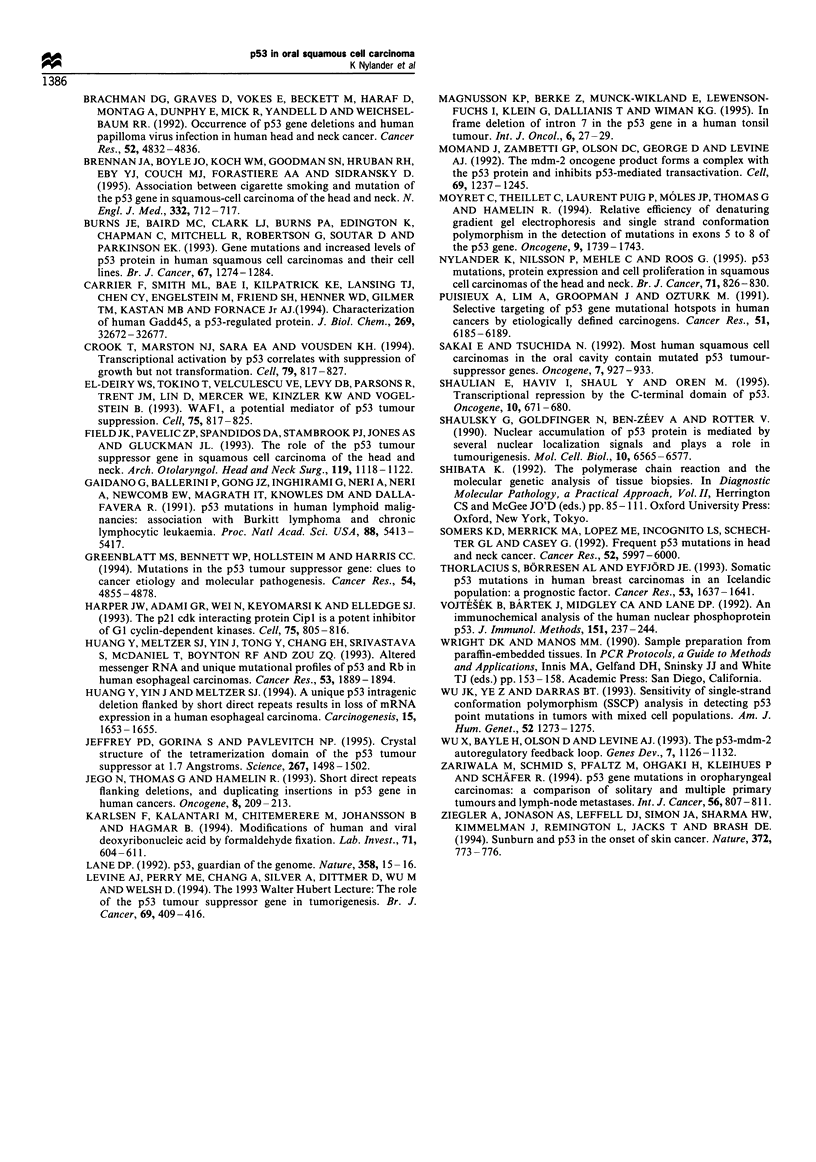# A non-random deletion in the p53 gene in oral squamous cell carcinoma.

**DOI:** 10.1038/bjc.1996.262

**Published:** 1996-06

**Authors:** K. Nylander, E. B. Schildt, M. Eriksson, A. Magnusson, C. Mehle, G. Roos

**Affiliations:** Department of Oral Pathology, Umeå University, Sweden.

## Abstract

**Images:**


British Journal of Cancer (1996) 73, 1381–1386
© 1996 Stockton Press All rights reserved 0007-0920/96 $12.00

### A non-random deletion in the *p53* gene in oral squamous cell carcinoma

K Nylander[1,2], EB Schildt[3], M Eriksson[3], A Magnusson[4], C Mehle[2] and G Roos[2]

*Departments of [1]Oral Pathology, [2]Pathology and [3]Oncology, Umeå University, S-901 87 Umeå, Sweden; [4]Department of Oncology, Örebro Medical Centre, S-701 85 Örebro, Sweden.*

**Summary** In a retrospective study of the mutational spectrum of the *p53* gene in oral squamous cell carcinoma, 80 primary tumours diagnosed in 1980–90 were included. Using polymerase chain reaction/single strand conformation polymorphism (PCR/SSCP) analysis 47 mutations were found distributed in 39 of the tumours (49%). Unexpectedly, the majority of the mutations (29/47; 62%) were found in exon 8, and at sequencing 17 of them showed a 14 bp deletion in codons 287–292, causing formation of a stop codon and accordingly a truncated protein lacking the C-terminal. The majority of the patients with the 14 bp deletion were women (13/17), and it seemed as though certain potential risk factors for carcinoma of the head and neck were less common in this group.

**Keywords:** *p53* gene; deletion; squamous cell carcinoma; head and neck; epidemiology

The p53 protein, encoded by the tumour-suppressor gene *p53*, encompasses three distinct regions: (1) the amino-terminus with a transactivation domain (codons 20–42); (2) the mid-region with a sequence-specific DNA binding domain (codons 100–293) and (3) the carboxy-terminus with a nuclear localisation domain (codons 316–325) and an oligomerisation domain (codons 319–360) (Greenblatt *et al.*, 1994). In the mid-region, four of the five evolutionary conserved domains are found. The wild-type p53 protein acts as a transcription factor capable of activating transcription of certain genes such as *mdm2* (Momand *et al.*, 1992; Wu *et al.*, 1993), *WAF1/Cip1* (El-Deiry *et al.*, 1993; Harper *et al.*, 1993) and *Gadd45* (Carrier *et al.*, 1994). *Mdm2* is an oncogene that exerts negative feedback on p53 (Momand *et al.*, 1992; Wu *et al.*, 1993), *WAF1/Cip1* is an inhibitor of cyclin-dependent kinases (El-Deiry *et al.*, 1993; Harper *et al.*, 1993) and *Gadd45* is capable of blocking progression through the cell cycle in $G_1$, a process reversible by *mdm2* (Levine *et al.*, 1994). p53 also controls the cell cycle as 'guardian of the genome' (Lane, 1992), and in UV-damaged skin it is considered 'guardian of the tissue' (Ziegler *et al.*, 1994) inducing apoptosis in sunburn cells.

Mutations in the p53 gene are frequent in human cancer, mostly affecting the conserved parts of the protein. The importance of p53 mutations in human tumours is, however, not fully understood. In breast carcinomas, p53 mutation has been suggested as an important indictaor of short-term survival (Thorlacius *et al.*, 1993), whereas p53 gene mutations and deletions so far have not shown any association with disease progression in squamous cell carcinoma of the head and neck (SCCHN) (Ahomadegbe *et al.*, 1995).

In SCCHN, mutations in the *p53* gene have been found in around 40–50% of the tumours (Boyle *et al.*, 1993; Brachman *et al.*, 1992; Brennan *et al.*, 1995; Nylander *et al.*, 1995).

Codons 238–248 and 278–281 have been pinpointed as hotspots for these mutations (Field *et al.*, 1993; Sakai and Tsuchida, 1992; Somers *et al.*, 1992). p53 mutations seem to be dependent on epidemiological factors, e.g. geographic origin which in hepatocellular carcinoma has been shown to be a factor affecting the mutational spectrum of p53 (Greenblatt *et al.*, 1994). The spectrum has also been found to vary between different countries and races in SCCHN. Thus, mutations have most frequently been found in exon 7 in the USA, whereas exon 8 was more frequently mutated in Japan (Field *et al.*, 1993; Sakai and Tsuchida, 1992; Somers *et al.*, 1992). On the whole, approximately 98% of all p53 mutations in SCCHN are found within exons 5–8 (Greenblatt *et al.*, 1994). The mutational pattern in SCCHN may also be influenced by exogenous carcinogens, such as mutagens in cigarette smoke, e.g. benzo(*a*)pyrene, that preferentially induce transversions (G:C→T:A) (Puisieux *et al.*, 1991). Furthermore, the spectrum of p53 mutations is wider in cigarette smokers who also drink alcohol, whereas *p53* mutations in non-smokers/non-drinkers predominate at CpG sites which are suggested as endogenous mutational 'hotspots' (Brennan *et al.*, 1995).

Mutations in the p53 gene are mainly of missense type, constituting up to 79–86% of all mutations (Greenblatt *et al.*, 1994; Levine *et al.*, 1994), whereas only approximately 8% of all mutations found are deletions and insertions (Levine *et al.*, 1994). In SCCHN deletions have, however, been found in a higher frequency constituting up to 30% of all mutations (Brachman *et al.*, 1992; Burns *et al.*, 1993). The obvious predominance of missense over non-missense mutations is, however, only seen between codons 130 and 286 (Greenblatt *et al.*, 1994). In an earlier study of p53 mutations in SCCHN we found a deletion of 14 bp in exon 8 in one tumour (Nylander *et al.*, 1995). Big deletions have been reported in other studies of SCCHN (Ahomadegbe *et al.*, 1995; Magnusson *et al.*, 1995; Zariwala *et al.*, 1994) as well as in other tumours, such as colon cancer, breast cancer, leukaemia and oesophageal carcinoma (Jego *et al.*, 1993; Huang *et al.*, 1994).

In the present expanded study we investigated the mutational spectrum of the p53 gene in 80 primary oral squamous cell carcinomas diagnosed in the period 1980–1990 in the northern part of Sweden. In this paper we report the finding of a specific 14 bp deletion in exon 8 that we correlated with epidemiological data collected from these patients.

### Materials and methods

### *Material*

Eighty primary oral squamous cell carcinomas diagnosed in the Departments of Oral Pathology and Pathology in Umeå, Sweden, during the period 1980–90 with representative formalin-fixed and paraffin-embedded samples were chosen for the study. The material was limited to tumours located in the oral cavity, excluding pharyngeal and lip carcinoma. Control samples consisted of DNA extracted from placenta.

### *DNA extraction*

On paraffin-embedded blocks of oral squamous cell carcinomas as much normal tissue as possible was scraped

Correspondence: K Nylander
Received 23 August 1995; revised 28 November 1995; accepted 13 December 1995

off with a scalpel. Depending on the size of the tumour, two to five 10-μm sections were cut from each sample. For each block a new microtome knife blade was used, and sections were put in sterile tubes using a tweezer which was washed in xylol between sectioning of each block. DNA was extracted according to Shibata (1992). In brief, sections were dewaxed in a series of xylene and ethanol and dried either at room temperature or in a speedvac (Savant Speedvac Plus, SC 110A). Extraction buffer consisting of 100 mM Tris and 1 mM EDTA, pH 8.0, was added together with proteinase K at a concentration of 400 μg $ml^{-1}$. Samples were incubated overnight at 37°C, and the following day boiled for 7 min and centrifuged, after which DNA was found in the supernatant.

For samples that did not amplify after 2–3 separate PCR reactions, new sections were cut, and after incubation in xylol and ethanol the dried pellet was incubated overnight at 37°C in an extraction buffer consisting of 50 mM Tris, 1 mM EDTA, 0.5% Tween 20 and 200 μg $ml^{-1}$ proteinase K (Wright and Manos, 1990).

*Primers*

Exons 5–9 of the p53 gene were amplified from each tumour using primers earlier characterised (Gaidano *et al.*, 1991). All primers were obtained from Scandinavian Gene Synthesis, Köping, Sweden.

*PCR reaction and SSCP analysis*

Each PCR reaction consisted of 1–2 μl of the DNA solution (DNA content not determined by spectrophotometry), 2.5 μM dNTPs, 10 pmol of each primer, 1 μCi of [α-$^{32}$P]dCTP (Amersham International, Bucks, UK), 1 × *Taq* polymerase buffer with 1.5 mM magnesium chloride, and 0.5 U of *Taq* polymerase (Boehringer Mannheim, Germany). The total reaction volume was 10 μl. After a 'hot start' at 94°C for 10 min, 35 cycles with denaturation at 94°C for 30 s, annealing at 55°C for 1 min and extension at 72°C for 1 min, were performed using a programmable thermal controller, PTC-100 (MJ Research, Watertown, MA, USA). The PCR reaction was finished at 72°C for 10 min.

An alternative PCR programme was applied for samples that had not amplified and were resectioned. In this programme a 'hot start' at 94°C for 10 min was followed by 40 cycles with denaturation at 94°C for 1 min, annealing at 55°C for 1 min and extension at 72°C for 2 min. The last cycle was followed by a final extension at 72°C for 5 min (Wright and Manos, 1990). The reason for prolonged times for denaturation and extension is that amplification from prepared paraffin-embedded tissue has been found to be less efficient than amplification of DNA from fresh tissue (Wright and Manos, 1990).

Reaction mixture (2 μl) was diluted with 50 μl 0.1% sodium dodecyl sulphate (SDS)/10 mM EDTA and 52 μl 98% formamide, 0.05% bromophenol blue, 0.05% xylene cyanol and 20 mM sodium hydroxide. This mixture was heated at 95°C for 5 min and chilled on ice, then 3 μl was loaded in each lane of a 6% polyacrylamide/TBE gel with 10% glycerol. Gels were run at room temperature at 3 W for 16–20 h, and autoradiography was performed at −70°C for 4–48 h.

*Cloning and sequencing of PCR products*

After the initial PCR/SSCP analysis of all tumours was finished a new non-radioactive PCR reaction was performed for tumours to be sequenced. This reaction amplifying the mutated exon was performed as described above with a higher concentration of dNTPs (200 μM) and a total volume of 50 μl. For ligation and cloning of the PCR product, a pGEM-T Vector System (Promega) was used, with a 1:1 molar ratio of insert–vector.

At least 16 and at most 48 different clones from each tumour were first analysed by PCR/SSCP, and then a minimum of two clones, showing the same mutation as was found in the initial PCR/SSCP analysis, were sequenced. Following plasmid preparation dideoxy sequencing of both strands was performed.

*PCR control reactions*

For tumours with mutation in exon 8 three different control PCR reactions were performed. The first reaction used new primers upstream and downstream of the initial primer pair. Primers used were: 5'CTGCCTCTTGCTTCTCTTTTC and 3'TGGTGTTGTTGGGCAGTG (Scandinavian Gene Synthesis). The PCR programme was the same as the one used before, except for an annealing temperature of 56°C. PCR products were pretreated and run on a gel as described earlier.

For the second control reaction new sections were cut from the paraffin blocks, and a new set of the same primers as was initially used for exon 8 was purchased as well as a new dNTP mixture and new *Taq* polymerase. The PCR products were analysed on a 6% polyacrylamide/TBE gel as described earlier. All steps from sectioning, DNA extraction to mixing of the PCR reaction were performed in another laboratory, which was not involved in our earlier p53 analysis.

To exclude the possibility of contamination of tumour samples by vector-ligated exon 8-specific DNA, a third control reaction was performed. In this non-radioactive PCR reaction comprising 30 cycles with denaturation at 94°C for 1 min, annealing at 56°C for 1 min and extension at 72°C for 2 min, primers specific for the pGEM-t vector, SP6 and T7 (Promega) were applied. The reaction volume consisted of 50 μl with 10 μl of DNA extract.

*Immunohistochemistry*

For the immunohistochemical analysis of p53 protein a monoclonal antibody, DO7 (Novocastra Laboratories, Newcastle, UK), recognising a denaturation-resistant epitope between amino acids 1 and 45 was applied (Vojtěšek *et al.*, 1992).

### Results

*p53 mutation analysis using PCR and SSCP*

Amplification of exon 5–8 was accomplished in 89–96% of the tumours, and of exon 9 in 60% of the tumours. A total of 47 mutations were found distributed in 39 tumours, as eight of these had two mutations in different exons. All tumours with two mutations were, with one exception, located either in the tongue or the floor of the mouth.

The majority of the mutations (29 out of 47; 62%) were found in exon 8, and many of those showed a concordant pattern in the SSCP analysis (Figure 1). The remaining 18 mutations were almost equally distributed between exons 5 (seven), 6 (four) and 7 (seven). No mutations were found in exon 9.

*Sequencing of mutations in exon 8*

The predominance of mutations in exon 8 was unexpected and made us investigate these tumours further, first by cloning and sequencing and then by different control PCR reactions. The majority of the sequenced tumours (17/29; 59%) showed a specific deletion of 14 bp in codons 287–292. This deletion caused a frameshift, formation of a stop codon near the end of the exon and accordingly a truncated protein. The same 14 bp deletion was also found in two other tumours, but only in a single clone among 32–48 clones that were scanned. These two tumours were only considered mutation positive in the SSCP analysis and not in the sequence analysis and were therefore not included in further analysis. Five of the eight tumours with two mutations had the 14 bp deletion plus a mutation in either exon 5, 6 or 7.

For details of the mutations in exon 8 see Table I and Figure 2.

*PCR control reactions*

For control reactions, a new set of primers external to the initial primer set was first applied, giving PCR products of up to 281 bp in length. In the following SSCP analysis no consistent difference in band pattern could be seen between tumour samples and normal control samples from placenta. The SSCP pattern for tumours with the 14 bp deletion was consistent, whereas two variable band patterns for the normal control samples were found, one of these concordant with the pattern for the 14 bp deletion. Out of the three tumours with a point mutation (C→T) in different codons mutation could be detected in one case due to aberrant band pattern, whereas the other two tumours showed no aberration. A reduction in mutation-induced mobility change is often found when the amplified product exceeds 180 bp (Moyret *et al.*, 1994). This could explain the difficulty we had in differentiating between these new products being up to 281 bp in length. Amplification of these long fragments could not be achieved in all tumours, possibly owing to the formaldehyde fixation (Karlsen *et al.*, 1994).

In another control series all steps from sectioning and DNA extraction to mixing new reactions with fresh ingredients were performed in another laboratory, giving the same pattern on SSCP gels as found in the initial analysis (Figure 3). PCR products from tumours with the 14 bp deletion gave two distinct bands on agarose gel compared with one band for normal samples and samples with other mutations (Figure 4).

Additional samples taken at the same time as the initial biopsy were available from five patients. When analysing these samples, six additional mutations were found. In one

**Figure 1** Initial PCR/SSCP analysis of exon 8 of the *p53* gene. Lane 1, non-mutated tumour; lane 2, tumour with a transversion A→G, codon 286 (tumour 15 in Table I); lane 3, tumour with 14 bp deletion, codons 287–292 (tumour 16 in Table I); lane 4, non-mutated tumour; lane 5, tumour with 14 bp deletion, codons 287–292 (tumour 17 in Table I).

**Figure 2** Distribution of 29 mutations in 27 tumours within exon 8. Each dot represents one mutation. Exact localisation of point mutations is given in the first row under the exon line and length of deletions in the second row. The 17 tumours with 14 bp deletion between codons 287–292 constitute group A in the statistical comparison with all other tumours (group B).

**Table I** Tumour localisation, sequence of p53 mutation and results from immunohistochemical analysis (IHC) for all tumours with mutation in exon 8.

| *No.* | *Localisation* | *Sequence* | *IHC* | *Smoker* | *Mutation in other exon* |
|---|---|---|---|---|---|
| 1 | Tongue | Deletion 14 bp, codon 287-292 | Pos. | – | |
| 2 | Gingiva | Deletion 14 bp, codon 287-292 | Pos. | – | |
| 3 | Tongue | Deletion 14 bp, codon 287-292 | Neg. | – | Exon 7 |
| 4 | Floor of mouth | Deletion 14 bp, codon 287-292 | Pos. | – | |
| 5 | Floor of mouth | Deletion 14 bp, codon 287-292 | Pos. | –** | |
| 6 | Floor of mouth | Deletion 14 bp, codon 287-292 | Neg. | – | |
| 7 | Floor of mouth | Deletion 4 bp, codon 269-270 | Pos. | + | Exon 7 |
| 8 | Gingiva | Deletion 14 bp, codon 287-292 | Neg. | + | Exon 6 |
| 9 | Tongue | Deletion 14 bp, codon 287-292 | Pos. | + | |
| 10 | Tongue | Deletion 14 bp, codon 287-292 | Pos. | – | Exon 6 |
| 11 | Gingiva | Deletion 14 bp, codon 287-292 | Neg. | –** | |
| 12 | Floor of mouth | C→T, codon 282 | Pos. | – | |
| 13 | Gingiva | Deletion 14 bp, codon 287-292 | Pos. | – | |
| 14 | Gingiva | Deletion 14 bp, codon 287-292; A→G, codon 304 | Neg. | ? | |
| 15 | Floor of mouth | A→G, codon 286 | Neg. | + | |
| 16 | Bucca | Deletion 14 bp, codon 287-292 | Pos. | – | |
| 17 | Floor of mouth | Deletion 14 bp, codon 287-292 | Neg. | – | Exon 7 |
| 18 | Floor of mouth | Deletion 14 bp, codon 287-292; A→G, codon 304 | Neg. | –** | |
| 19 | Floor of mouth | C→T, codon 306 | Neg. | – | |
| 20* | Floor of mouth | Deletion 14 bp, codon 287-292 | Pos. | – | Exon 5 |
| 21 | Floor of mouth | C→T, codon 276 | Pos. | – | |
| 22* | Floor of mouth | Deletion 14 bp, codon 287-292 | Pos. | ? | |
| 23 | Gingiva | C→T, codon 306 | Neg. | + | |
| 24 | Floor of mouth | T→G, codon 270 | Neg. | + | |
| 25 | Tongue | T→G, codon 270 | Pos. | –** | Exon 5 |
| 26 | Gingiva | C→T, codon 282 | Pos. | + | |
| 27 | Floor of mouth | C→T, codon 282 | Pos. | –** | |

*Owing to technical difficulties only one of two clones from tumours 20 and 22 could be successfully sequenced. **Non-smokers who stopped smoking 10-30 years ago. ? Persons who refused to answer the questionnaire.

patient a precancerous lesion taken from the same location as the biopsy later diagnosed as carcinoma, most probably from the border area, was also analysed, showing a mutation in exon 8.

**Figure 3** PCR/SSCP control of exon 8 for tumours with a 14 bp deletion in codons 287–292 confirmed by sequencing. All steps in this control PCR reaction were performed in another laboratory, and new sections as well as new ingredients were used. Lane 1, tumour with the 14 bp deletion and a transition A→G, codon 304 (tumour 14 in Table I). The presence of two mutations in this tumour explains why the lowest band is not co-localised with the corresponding band in the other tumours with the 14 bp deletion; lane 2, tumour with the 14 bp deletion (tumour 4 in Table I); lane 3, new sections from the same tumour as in lane 2, showing the same pattern with somewhat weaker aberrant bands. This is explained by the fact that this part of the sample contained less tumour tissue; lane 4, tumour with the 14 bp deletion (tumour 17 in Table I). The initial analysis of the same tumour is shown in lane 5 in Figure 1; lane 5, normal placenta sample.

**Figure 4** Products from a non-radioactive PCR from three different tumours run on a 4% agarose gel. Lane 1, tumour with the 14 bp deletion showing two bands (tumour 4 in Table I); lane 2, tumour without the 14 bp deletion showing only one band; lane 3, tumour with the 14 bp deletion showing two bands (tumour 3 in Table I).

The third PCR control reaction with vector specific primers showed no product in any tumour sample tested, whereas products were seen in control samples consisting of amplified exon 7-specific DNA from the *p53* gene inserted into a pGEM-T vector (Figure 5).

*Epidemiology and statistical analysis*

Since all patients were included in an epidemiological case–control study, data on demographics and exposure conditions collected by questionnaires were available. Tumours were divided into two groups: group A including tumours with the 14 bp deletion (17 patients) and group B including all other tumours (63 patients). Five patients, however, had refused to answer the questionnaire and the groups were therefore reduced to 15 and 60 patients respectively.

There was a statistically significant overrepresentation of women in group A (80%) compared with group B (47%). The majority of patients in group A (60%) were over 70 years at diagnosis compared with only 47% in group B. Patients in group A also showed significantly lower exposure to different chemical agents as well as a lower rate of tobacco smoking. The predominance of women in this group could,

**Figure 5** A non-radioactive PCR reaction with vector specific primers, SP6 and T7. Lane 1, size marker $\phi \times 174$; lane 3 and 4, positive controls consisting of amplified exon 7-specific DNA ligated into a pGEM-T vector; lane 5, negative control; lane 6 and 7, non-mutated tumours; lane 8, tumour with a transversion A→G, codon 286 (tumour 15 in Table I); lane 9–13, tumours with the 14 bp deletion, codons 287–292; lane 15, size marker pGEM.

**Table II** Occurrence (%) of different factors in group A (with the 14 bp deletion) and group B (all other tumours), and *P*-values based on chi-square test according to Pearson for the difference between the groups

| | *Group A* (n=*15*) | *Group B* (n=*60*) | P-*value* |
|---|---|---|---|
| Demographic factors | | | |
| Female | 80.0 | 46.7 | 0.02 |
| Dead at latest follow-up | 80.0 | 60.0 | 0.15 |
| Age over 70 at diagnosis | 60.0 | 46.7 | 0.36 |
| Exposure factors | | | |
| Chemical agents at work | 0.0 | 38.3 | 0.004 |
| Organic solvents | 0.0 | 18.3 | 0.073 |
| Pesticides | 0.0 | 13.3 | 0.13 |
| Tobacco smoking | 33.3 | 55.0 | 0.13 |
| Dental radiograph | 38.5 | 66.0 | 0.07 |
| Gold fillings | 11.1 | 36.7 | 0.13 |
| Amalgam fillings | 36.4 | 72.5 | 0.02 |
| Number of patients | 15 | 60 | |

however, be a confounding factor when considering these exposures. Other investigated exposure factors, with as yet unknown impact on the risk for SCCHN that differed between the groups were dental radiograph and gold and amalgam fillings (Table II).

The *P*-value for differences between group A and B was calculated using the chi-square test according to Pearson.

### Discussion

The aim of the present study was to investigate the mutational spectrum in the *p53* gene in a group of oral squamous cell carcinoma. The predominance of mutations in exon 8 was an unexpected finding. Predominance of mutations in one exon is consistent with earlier results where a geographically based overrepresentation of *p53* mutations in different exons has been found (Field *et al.*, 1993; Sakai and Tsuchida, 1992; Somers *et al.*, 1992). Almost two-thirds (63%) of the mutations found in exon 8 were deletions of 14 bp in codons 287–292. Big deletions occurring in the *p53* gene have been reported previously (Ahomadegbe *et al.*, 1995; Huang *et al.*, 1994), but not to such an extent as in this material, constituting over a third of all mutations found. These deletions were located outside the five highly conserved regions between codons 13–280. For SCCHN codons 287–292 is not a previously known hotspot area for mutations, but in a study of oesophageal tumours (Huang *et al.*, 1993) a deletion of 5 bp in the same area has been reported.

When using a sensitive method like PCR, the question of contamination as an explanation for the finding of a novel deletion naturally arises. However, in the present study extraordinary care was taken throughout the entire work to avoid any possibility of contamination. At sectioning a much more careful method than generally recommended (Shibata, 1992) was applied, and in the control PCR reaction performed in an outside laboratory only fresh ingredients (including primers) were used. Furthermore, no amplifying or cloning of the exon 8-specific DNA was performed until all tumours had been analysed by PCR/SSCP, avoiding any possibility of contamination of tumour samples or PCR ingredients. Control of tumour samples for vector contamination also gave a negative result. Some blocks were also resectioned at a later time point showing concordant results in the analysis. In this control reaction all ingredients were new, and all steps performed in a laboratory not before used for PCR analysis or cloning. The non-existence of the same 14 bp deletion in samples from ulcerative colitis and colon carcinoma run at the same time using the same equipment and ingredients furthermore contradicts any possible contamination and confirms the true nature of this novel deletion in oral squamous cell carcinoma.

One explanation for the 14 bp deletion could be the close proximity of three GA dinucleotides in the area, one located just outside the deletion and the other two at each end of the deletion. This might cause erroneous reannealing between the first repeat and the complementary strand of the third repeat when the DNA chain opened before replication (Jego *et al.*, 1993). Thus, a loop consisting of the two other GA dinucleotides and the sequence in between was formed and excised before actual replication occurred.

The primary effect of the 14 bp deletion is the formation of a stop codon, and accordingly a truncated protein with loss of the C-domain containing the oligomerisation domain and the nuclear localisation signals (NLS). *In vitro* tetramerisation is not essential for DNA binding of p53, whereas at least dimerisation is necessary for p53 activity *in vivo* (Jeffrey *et al.*, 1995). For retaining the transactivational activity the location of the truncation is of importance (Crook *et al.*, 1994). Appreciable DNA binding and transactivation has been found after deletion of up to 87 residues from the C-terminal (Shaulian *et al.*, 1995), near truncation caused by the 14 bp deletion. The mutation we found could theoretically cause production of a p53 protein incapable of transactivation accumulating in the cytoplasm, as it is lacking the NLS. A protein lacking the NLS is, however, not ultimately located in the cytoplasm, as wild-type p53 devoid of NLS *in vitro* has been shown to complex with another nuclear protein, and thereby entered the nucleus (Shaulsky *et al.*, 1990). Immunohistochemical staining of the tumours in this material with an antibody against p53 showed nuclear as well as cytoplasmic staining in three of the tumours with the 14 bp deletion. Cytoplasmic staining was, however, also found in four other tumours, three without any mutation at all and one with a mutation in exon 6. This makes it less probable that the p53 protein found in the cytoplasm of tumours with the deletion was accumulated truncated protein. In the immunohistochemical analysis 10 of the 17 tumours with the 14 bp deletion showed a positive staining, whereas seven tumours were negative. This can be explained by the sensitivity of the PCR/SSCP technique by which mutations can be detected with an admixture of up to 85–95% normal non-mutated cells (Wu JK *et al.*, 1993). Accordingly wild-type p53 protein or wild-type p53 protein bound to another protein found in some tumour cells or in normal admixing cells could cause a negative or positive staining respectively.

In conclusion, in this study a new non-random deletion in exon 8 in oral squamous cell carcinomas was found. The validity of this finding was confirmed by extensive control experiments. The cause and clinical significance of this new 14 bp deletion is so far not known. Of patients with the deletion 80% were women, and there was a tendency for later onset of the disease. In order to characterise this new group of patients as thoroughly as possible, exposure to different factors was investigated. There is, to our knowledge, no scientific basis for the association between dental treatment and p53 mutations, but the deletion group differed significantly from the other patients concerning filling material. Our data for this group furthermore implicated a possible independence of known risk factors for SCCHN, such as smoking.

**Acknowledgements**
We are grateful to Ms Britta Lindgren for immunohistochemical staining and sectioning of all the samples. This study was supported by grants from the Lion's Cancer Research Foundation, Umeå University, Sahlberg's Fund, Umeå University, the Swedish Cancer Society, the Swedish Tobacco Company, Swedish Society for Medical Research and the Swedish Dental Society.

### References

AHOMADEGBE JC, BARROIS M, FOGEL S, LE BIHAN ML, DOUCRACY S, DUVILLARD P, ARMAND JP AND RIOU G. (1995). High incidence of p53 alterations (mutation, deletion, overexpression) in head and neck primary tumours and metastases; absence of correlation with clinical outcome. Frequent protein overexpression in normal epithelium and in early noninvasive lesions. *Oncogene*, **10**, 1217–1227.

BOYLE JO, HAKIM J, KOCH W, VAN DER RIET P, HRUBAN RH, ROA RA, CORREO R, EBY YJ, RUPPERT JM AND SIDRANSKY D. (1993). The incidence of p53 mutations increases with progression of head and neck cancer. *Cancer Res.*, **53**, 4477–4480.

BRACHMAN DG, GRAVES D, VOKES E, BECKETT M, HARAF D, MONTAG A, DUNPHY E, MICK R, YANDELL D AND WEICHSELBAUM RR. (1992). Occurrence of p53 gene deletions and human papilloma virus infection in human head and neck cancer. *Cancer Res.*, **52**, 4832–4836.

BRENNAN JA, BOYLE JO, KOCH WM, GOODMAN SN, HRUBAN RH, EBY YJ, COUCH MJ, FORASTIERE AA AND SIDRANSKY D. (1995). Association between cigarette smoking and mutation of the p53 gene in squamous-cell carcinoma of the head and neck. *N. Engl. J. Med.*, **332**, 712–717.

BURNS JE, BAIRD MC, CLARK LJ, BURNS PA, EDINGTON K, CHAPMAN C, MITCHELL R, ROBERTSON G, SOUTAR D AND PARKINSON EK. (1993). Gene mutations and increased levels of p53 protein in human squamous cell carcinomas and their cell lines. *Br. J. Cancer*, **67**, 1274–1284.

CARRIER F, SMITH ML, BAE I, KILPATRICK KE, LANSING TJ, CHEN CY, ENGELSTEIN M, FRIEND SH, HENNER WD, GILMER TM, KASTAN MB AND FORNACE Jr AJ.(1994). Characterization of human Gadd45, a p53-regulated protein. *J. Biol. Chem.*, **269**, 32672–32677.

CROOK T, MARSTON NJ, SARA EA AND VOUSDEN KH. (1994). Transcriptional activation by p53 correlates with suppression of growth but not transformation. *Cell*, **79**, 817–827.

EL-DEIRY WS, TOKINO T, VELCULESCU VE, LEVY DB, PARSONS R, TRENT JM, LIN D, MERCER WE, KINZLER KW AND VOGELSTEIN B. (1993). WAF1, a potential mediator of p53 tumour suppression. *Cell*, **75**, 817–825.

FIELD JK, PAVELIC ZP, SPANDIDOS DA, STAMBROOK PJ, JONES AS AND GLUCKMAN JL. (1993). The role of the p53 tumour suppressor gene in squamous cell carcinoma of the head and neck. *Arch. Otolaryngol. Head and Neck Surg.*, **119**, 1118–1122.

GAIDANO G, BALLERINI P, GONG JZ, INGHIRAMI G, NERI A, NERI A, NEWCOMB EW, MAGRATH IT, KNOWLES DM AND DALLA-FAVERA R. (1991). p53 mutations in human lymphoid malignancies: association with Burkitt lymphoma and chronic lymphocytic leukaemia. *Proc. Natl Acad. Sci. USA*, **88**, 5413–5417.

GREENBLATT MS, BENNETT WP, HOLLSTEIN M AND HARRIS CC. (1994). Mutations in the p53 tumour suppressor gene: clues to cancer etiology and molecular pathogenesis. *Cancer Res.*, **54**, 4855–4878.

HARPER JW, ADAMI GR, WEI N, KEYOMARSI K AND ELLEDGE SJ. (1993). The p21 cdk interacting protein Cip1 is a potent inhibitor of G1 cyclin-dependent kinases. *Cell*, **75**, 805–816.

HUANG Y, MELTZER SJ, YIN J, TONG Y, CHANG EH, SRIVASTAVA S, McDANIEL T, BOYNTON RF AND ZOU ZQ. (1993). Altered messenger RNA and unique mutational profiles of p53 and Rb in human esophageal carcinomas. *Cancer Res.*, **53**, 1889–1894.

HUANG Y, YIN J AND MELTZER SJ. (1994). A unique p53 intragenic deletion flanked by short direct repeats results in loss of mRNA expression in a human esophageal carcinoma. *Carcinogenesis*, **15**, 1653–1655.

JEFFREY PD, GORINA S AND PAVLEVITCH NP. (1995). Crystal structure of the tetramerization domain of the p53 tumour suppressor at 1.7 Angstroms. *Science*, **267**, 1498–1502.

JEGO N, THOMAS G AND HAMELIN R. (1993). Short direct repeats flanking deletions, and duplicating insertions in p53 gene in human cancers. *Oncogene*, **8**, 209–213.

KARLSEN F, KALANTARI M, CHITEMERERE M, JOHANSSON B AND HAGMAR B. (1994). Modifications of human and viral deoxyribonucleic acid by formaldehyde fixation. *Lab. Invest.*, **71**, 604–611.

LANE DP. (1992). p53, guardian of the genome. *Nature*, **358**, 15–16.

LEVINE AJ, PERRY ME, CHANG A, SILVER A, DITTMER D, WU M AND WELSH D. (1994). The 1993 Walter Hubert Lecture: The role of the p53 tumour suppressor gene in tumorigenesis. *Br. J. Cancer*, **69**, 409–416.

MAGNUSSON KP, BERKE Z, MUNCK-WIKLAND E, LEWENSON-FUCHS I, KLEIN G, DALLIANIS T AND WIMAN KG. (1995). In frame deletion of intron 7 in the p53 gene in a human tonsil tumour. *Int. J. Oncol.*, **6**, 27–29.

MOMAND J, ZAMBETTI GP, OLSON DC, GEORGE D AND LEVINE AJ. (1992). The mdm-2 oncogene product forms a complex with the p53 protein and inhibits p53-mediated transactivation. *Cell*, **69**, 1237–1245.

MOYRET C, THEILLET C, LAURENT PUIG P, MÓLES JP, THOMAS G AND HAMELIN R. (1994). Relative efficiency of denaturing gradient gel electrophoresis and single strand conformation polymorphism in the detection of mutations in exons 5 to 8 of the p53 gene. *Oncogene*, **9**, 1739–1743.

NYLANDER K, NILSSON P, MEHLE C AND ROOS G. (1995). p53 mutations, protein expression and cell proliferation in squamous cell carcinomas of the head and neck. *Br. J. Cancer*, **71**, 826–830.

PUISIEUX A, LIM A, GROOPMAN J AND OZTURK M. (1991). Selective targeting of p53 gene mutational hotspots in human cancers by etiologically defined carcinogens. *Cancer Res.*, **51**, 6185–6189.

SAKAI E AND TSUCHIDA N. (1992). Most human squamous cell carcinomas in the oral cavity contain mutated p53 tumour-suppressor genes. *Oncogene*, **7**, 927–933.

SHAULIAN E, HAVIV I, SHAUL Y AND OREN M. (1995). Transcriptional repression by the C-terminal domain of p53. *Oncogene*, **10**, 671–680.

SHAULSKY G, GOLDFINGER N, BEN-ZÉEV A AND ROTTER V. (1990). Nuclear accumulation of p53 protein is mediated by several nuclear localization signals and plays a role in tumourigenesis. *Mol. Cell. Biol.*, **10**, 6565–6577.

SHIBATA K. (1992). The polymerase chain reaction and the molecular genetic analysis of tissue biopsies. In *Diagnostic Molecular Pathology, a Practical Approach, Vol. II*, Herrington CS and McGee JO'D (eds.) pp. 85–111. Oxford University Press: Oxford, New York, Tokyo.

SOMERS KD, MERRICK MA, LOPEZ ME, INCOGNITO LS, SCHECHTER GL AND CASEY G. (1992). Frequent p53 mutations in head and neck cancer. *Cancer Res.*, **52**, 5997–6000.

THORLACIUS S, BÖRRESEN AL AND EYFJÖRD JE. (1993). Somatic p53 mutations in human breast carcinomas in an Icelandic population: a prognostic factor. *Cancer Res.*, **53**, 1637–1641.

VOJTĚŠEK B, BÁRTEK J, MIDGLEY CA AND LANE DP. (1992). An immunochemical analysis of the human nuclear phosphoprotein p53. *J. Immunol. Methods*, **151**, 237–244.

WRIGHT DK AND MANOS MM. (1990). Sample preparation from paraffin-embedded tissues. In *PCR Protocols, a Guide to Methods and Applications*, Innis MA, Gelfand DH, Sninsky JJ and White TJ (eds.) pp. 153–158. Academic Press: San Diego, California.

WU JK, YE Z AND DARRAS BT. (1993). Sensitivity of single-strand conformation polymorphism (SSCP) analysis in detecting p53 point mutations in tumors with mixed cell populations. *Am. J. Hum. Genet.*, **52** 1273–1275.

WU X, BAYLE H, OLSON D AND LEVINE AJ. (1993). The p53-mdm-2 autoregulatory feedback loop. *Genes Dev.*, **7**, 1126–1132.

ZARIWALA M, SCHMID S, PFALTZ M, OHGAKI H, KLEIHUES P AND SCHÄFER R. (1994). p53 gene mutations in oropharyngeal carcinomas: a comparison of solitary and multiple primary tumours and lymph-node metastases. *Int. J. Cancer*, **56**, 807–811.

ZIEGLER A, JONASON AS, LEFFELL DJ, SIMON JA, SHARMA HW, KIMMELMAN J, REMINGTON L, JACKS T AND BRASH DE. (1994). Sunburn and p53 in the onset of skin cancer. *Nature*, **372**, 773–776.